# Caspase-3 knockout inhibits intervertebral disc degeneration related to injury but accelerates degeneration related to aging

**DOI:** 10.1038/s41598-019-55709-3

**Published:** 2019-12-18

**Authors:** Takashi Ohnishi, Katsuhisa Yamada, Koji Iwasaki, Takeru Tsujimoto, Hideaki Higashi, Taichi Kimura, Norimasa Iwasaki, Hideki Sudo

**Affiliations:** 1 Department of Orthopedic Surgery, Faculty of Medicine and Graduate of Medicine, Hokkaido University, Sapporo, Hokkaido Japan; 2 Department of Advanced Medicine for Spine and Spinal Cord Disorders, Faculty of Medicine and Graduate of Medicine, Hokkaido University, Sapporo, Hokkaido Japan; 3 Research Center for Zoonosis Control, Hokkaido University, Sapporo, Hokkaido Japan; 4 Department of Pathological Diagnosis, Hokkaido Medical Center, Sapporo, Hokkaido Japan

**Keywords:** Apoptosis, Osteoarthritis

## Abstract

Approximately 40% of people under 30 and over 90% of people 55 or older suffer from moderate-to-severe levels of degenerative intervertebral disc (IVD) disease in their lumbar spines. Surgical treatments are sometimes effective; however, the treatment of back pain related to IVD degeneration is still a challenge; therefore, new treatments are necessary. Apoptosis may be important in IVD degeneration because suppressing cell apoptosis inside the IVD inhibits degeneration. Caspase-3, the primary effector of apoptosis, may be a key treatment target. We analyzed caspase-3’s role in two different types of IVD degeneration using caspase-3 knockout (Casp-3 KO) mice. Casp-3 KO delayed IVD degeneration in the injury-induced model but accelerated it in the age-induced model. Our results suggest that this is due to different pathological mechanisms of these two types of IVD degeneration. Apoptosis was suppressed in the IVD cells of Casp-3 KO mice, but cellular senescence was enhanced. This would explain why the Casp-3 KO was effective against injury-induced, but not age-related, IVD degeneration. Our results suggest that short-term caspase-3 inhibition could be used to treat injury-induced IVD degeneration.

## Introduction

Approximately 40% of people under 30 and over 90% of people 55 years or older suffer from moderate-to-severe levels of degenerative intervertebral disc (IVD) disease in their lumbar spines^1^. The increasing incidence of IVD degeneration with age and its correlation with lower back pain, IVD herniation, and spinal canal stenosis is an alarming trend in modern society. It is the primary cause of disability for people younger than 45, with financial and emotional effects that cause severe strain^1^. Surgical treatments, including discectomy or fusion, are effective; however, several complications have been reported, including relapse of herniation^2^ and adjacent segment disease^3,4^, among others^5–7^. Accordingly, these treatments are compensating for the dysfunctions without solving the problem. In addition, the treatment of back pain related to IVD degeneration with surgical treatments is still a challenge. Therefore, a novel and fundamental approach to treating IVD degeneration is needed^7^.

Dysfunction is caused by degeneration in the IVDs subsequent to several factors, including injury^8,9^, aging^10,11^, and mechanical overload^5,12,13^. Furthermore, apoptosis plays a vital role in reducing the disc cell number during degeneration, leading to diminished generation, organization, and repair of the extracellular matrix^7,14^. Previous studies have provided evidences for IVD-cell apoptosis in the following species: humans^15^, mice by annular puncture^16^, and rats by mechanical loading^17^. Therefore, one possible approach to preventing IVD degeneration is inhibiting apoptosis^7,14,18^.

Numerous molecules are involved in apoptotic cascades, but the key executioners are caspases, a family of cysteine proteases capable of cleaving essential cellular substrates with aspartate residues^7^. Caspase-3, the effector caspase with the most significant effect, is localized downstream in the caspase cascade and is the primary effector apoptosis molecule^7^. We previously studied disc cell apoptosis inhibition via a caspase-3 knockdown as a treatment target for IVD degeneration in rabbits^7,12^. We showed that the caspase-3 knockdown in rabbit IVD cells prevented apoptosis, thus reducing injury-induced IVD degeneration^7^. In this study, we used a needle puncture to induce IVD degeneration in rabbits^1^. Injury-induced IVD degeneration has been described in previous studies wherein increased gene expression of type I and II collagen, fibronectin, several metalloproteinases (MMP-1, -9, and -13), various growth factors (fibroblast growth factor and transforming growth factor-alpha), pro-inflammatory cytokines [tumor necrosis factor-alpha and interleukin-1 beta (IL-1β)]^19,20^, and the coexpression of FasL with Fas induce the apoptosis of disc cells^21^. However, we recently identified histological differences between IVD degenerations caused by injury and caused by aging, which suggests there are pathological differences between these types of dysfunction^22,23^, resulting in different responses to treatment. In addition, because of its essential role in apoptosis, chronic inhibition of caspase-3 signaling may disrupt IVD homeostasis or tumorigenesis.

In addition, cellular senescence has been indicated as a pathology of age-related spontaneous IVD degeneration in previous studies^24–26^. The accumulation of senescent cells^25,26^ and increased gene expression of the degrading enzymes, namely, matrix metalloproteinase-13 (MMP-13) and aggrecanase [a disintegrin and metalloproteinase with thrombospondin motifs 5 (ADAMTS-5)]^24^ were indicated as the pathology. Therefore, an age-related spontaneous IVD degeneration model needs to be studied.

The present study’s objectives were to assess whether the different pathological mechanisms in the two types of IVD degeneration affect the response to caspase-3 deletion or cause any adverse effects related to chronic caspase-3 inhibition, mostly tumorigenesis. To this end, we compared IVD degeneration induced by injury and aging in caspase-3 knockout (Casp-3 KO) mice and wild-type (WT) mice. We also evaluated the existence of tumorigenesis histologically. We analyzed the caspase-3 gene’s role in IVD degeneration using Casp-3 KO mice and compared IVD degeneration induced by injury^3^ and aging^22,23^ to determine which type of IVD degeneration can be targeted by an anti-apoptotic strategy. Our study revealed that Casp-3 KO delayed IVD degeneration induced by injury, but accelerated degeneration induced by aging. Apoptosis was found to be the primary driving factor in injury-related IVD degeneration (IRD), whereas cellular senescence seemed to be the major factor driving age-related IVD degeneration (ARD). The present findings confirm the need to develop IVD degeneration treatment according to the cause.

## Results

### Casp-3 KO mice present smaller IVDs than WT mice but present higher cell density with age

To study caspase-3’s role in IVD degeneration, we constructed Casp-3 KO mice (Supplementary Fig. S1a). WT and Casp-3 KO mice were selected from two sets of littermates born from the same pair of heterozygotes. Analysis of whole-body skeletons in newborn Casp-3 KO mice revealed no apparent abnormalities (Supplementary Fig. S1b). The body weights of Casp-3 KO mice were lower than those of WT mice for 3 weeks to approximately 10 weeks after birth and were then identical until 14 weeks (Supplementary Fig. S1c). However, at 14 and 18 months old, Casp-3 KO mice had significantly lower body weights than WT mice (Supplementary Fig. S1d).

**Figure 1:**
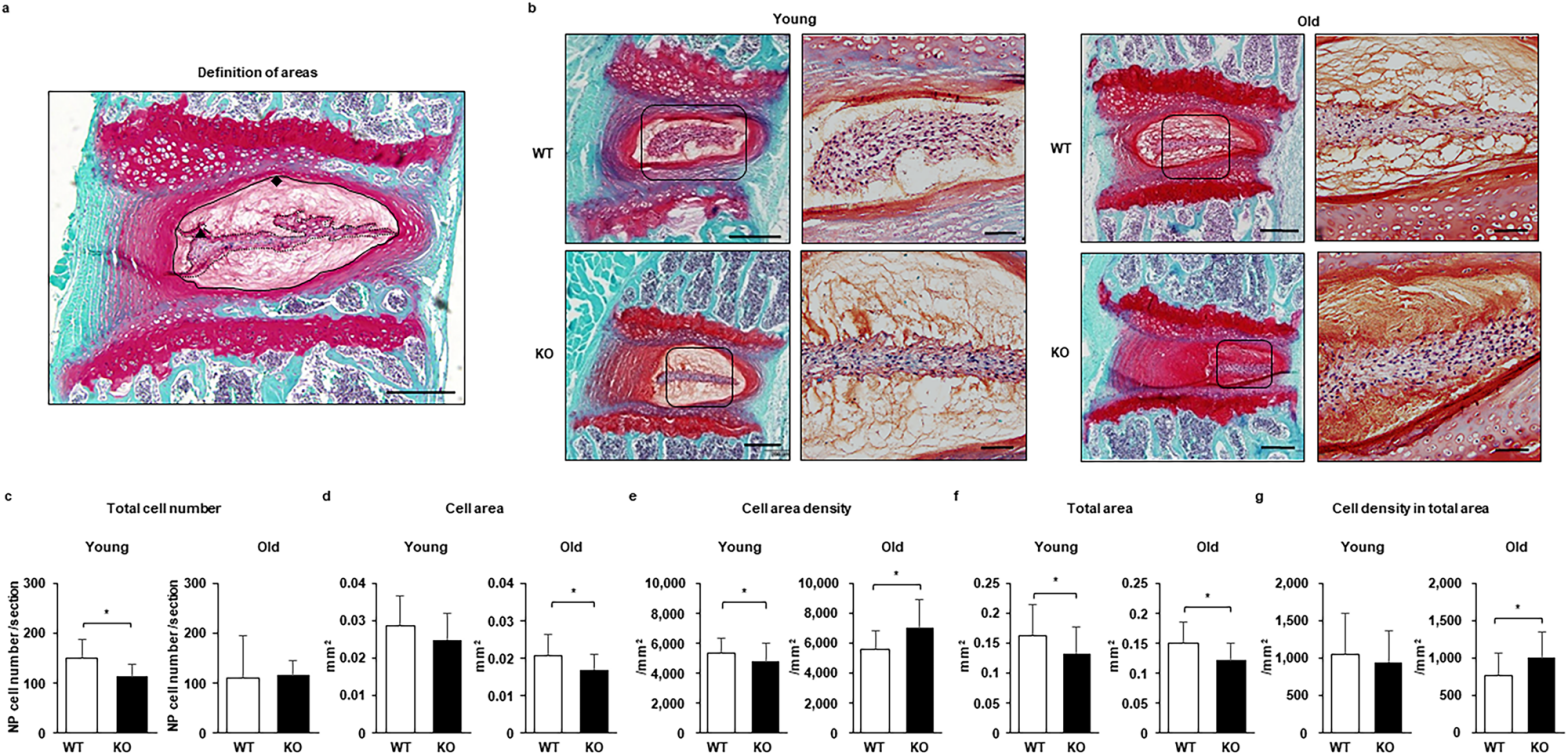
Caspase-3 knockout (KO) mice present a smaller nucleus pulposus (NP) than wild-type (WT) mice but exhibit a higher cell density with age. The sections were stained using Safranin O and fast green. (**a**) The definition of cell area (▲) and total area (◆) are shown. Scale bar, 200 μm. (**b**) Representative images from young (6-month-old) and old (14-month-old) WT and KO mice. Scale bars, 200 μm; 50 μm in magnified images. (**c**–**g**) Quantification of the total cell number (**c**), cell area (**d**), cell area density (**e**), total area (**f**), and cell density in the total area (**g**). The cells were counted, and the tissue areas were measured manually using Image J, version 1.47. Twenty-seven intervertebral discs (IVDs) from six mice were evaluated for young WT and KO mice, respectively. Thirty-five IVDs from six old WT mice and 36 IVDs from six old KO mice were assessed. Data are the mean ± the standard deviation (SD; *p < 0.05).

**Figure 2:**
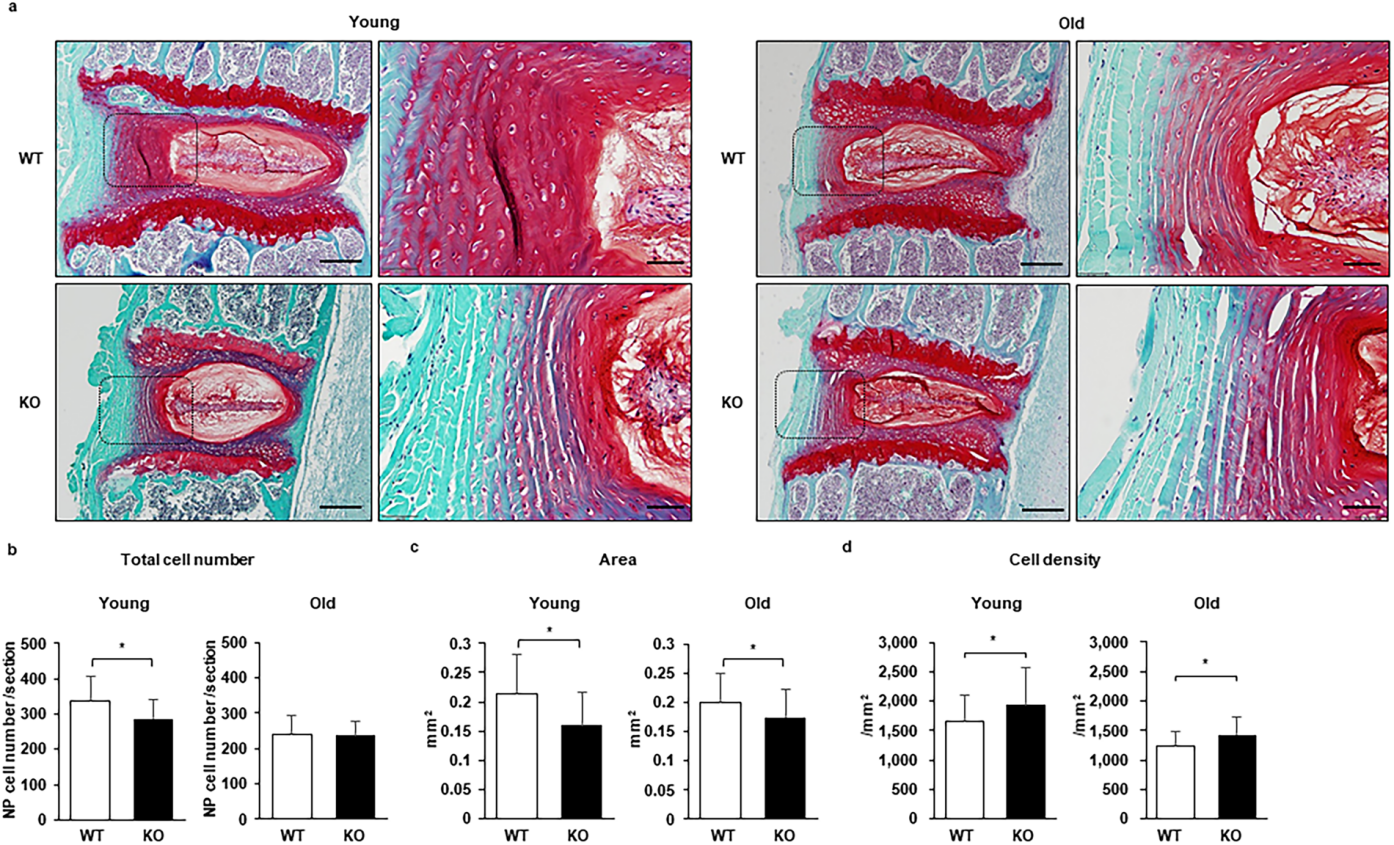
Caspase-3 KO mice presented smaller annulus fibrosus (AF) than WT mice but showed a higher cell density with age. The sections were stained with safranin O and fast green. (**a**) Representative images from young (6-month-old) and old (14-month-old) WT and KO mice. Scale bars, 200 μm; 50 μm in magnified images. (**b**–**d**) The quantification of the total cell number (**b**), area (**c**), and cell density (**d**). The cells were counted, and the tissue areas were measured manually using Image J, version 1.47. Twenty-seven IVDs from six young WT mice, 30 IVDs from six young KO mice, and 36 IVDs from six old WT and KO mice were evaluated. Data are the mean ± SD (*p < 0.05).

The IVDs are composed by the nucleus pulposus (NP) and annulus fibrosus (AF). The total cell numbers in the NP (Fig. 1a–c) and AF (Fig. 2a,b) of Casp-3 KO mice were lower than those of WT mice when the mice were young (six months old) but became identical in old (14 months old) mice. The tissue areas for the NP (Fig. 1a,b,f) and AF (Fig. 2a,c) were smaller in the Casp-3 KO mice than the WT mice regardless of age. In contrast, cell densities in the cell area of NP (Fig. 1a,b,e) and total NP area (Fig. 1a,b,g) were higher in old Casp-3 KO mice than in WT mice due to the smaller tissue area. In young mice, however, cell area density (Fig. 1a,b,e) and cell density in total area (Fig. 1a,b,g) were lower in the NP and higher in the AF (Fig. 2a,d) in Casp-3 KO mice than in WT mice.

**Figure 3:**
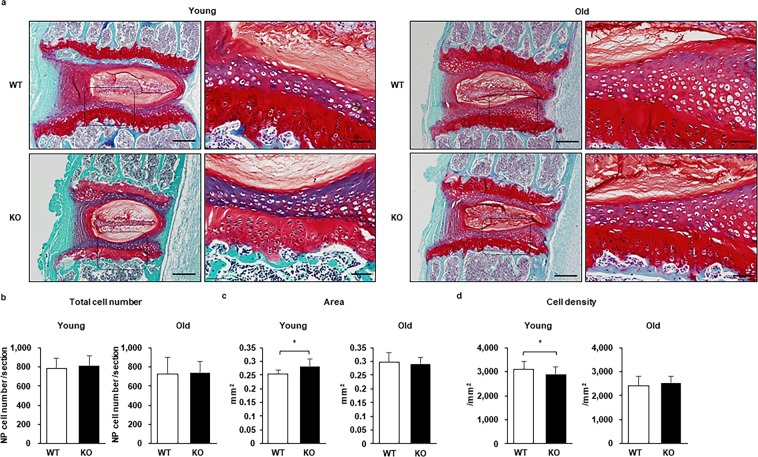
Caspase-3 KO mice presented a larger endplate (EP) but had a lower cell density than WT mice when young and became similar with age. The sections were stained with safranin O and fast green. (**a**) Representative images from young (6-month-old) and old (14-month-old) WT and KO mice. Scale bars, 200 μm; 50 μm in magnified images. (**b**–**d**) The quantification of the total cell number (**b**), area (**c**), and cell density (**d**). The cells were counted, and the tissue areas were measured manually using Image J, version 1.47. Twenty-seven IVDs from six young WT mice, 30 IVDs from six young KO mice, and 36 IVDs from six old WT and KO mice were evaluated. Data are the mean ± SD (*p < 0.05).

The endplate (EP), a tissue adjacent tissue to the IVD, was also analyzed as it is an essential structure in nutrition transport for the IVD^27^. No significant differences were found in the total cell numbers between WT and Casp-3 KO mice in both young and old populations (Fig. 3a,b). The EP tissue area was larger in Casp-3 KO mice than in WT mice when young, but no significant difference was found in old Casp-3 KO and WT mice (Fig. 3a,c). Consequently, the cell density was lower in young Casp-3 KO mice than in young WT mice due to the larger EP area in Casp-3 KO mice (Fig. 3a,c,d). The cell densities were similar between old WT and Casp-3 KO mice (Fig. 3a,d).

In summary, the Casp-3 KO resulted in smaller bodies and IVDs of the mice. It also resulted in higher cell densities in the IVDs, but a larger tissue area with lower cell densities in the cartilage of EPs.

### IVD cells of Casp-3 KO mice show resistance to apoptosis *in vitro* and slower injury-related IVD degeneration *in vivo*

Next, we investigated the apoptosis rate in IVDs of WT and Casp-3 KO mice. We focused on NP cells because NP cell apoptosis was evident in degenerated herniated NP in humans^28,29^, but AF cell apoptosis was only shown in an *in vitro* model. Our previous study also showed more drastic changes were seen histologically in the NP, compared to the AF, in the injury model^22^.

**Figure 4:**
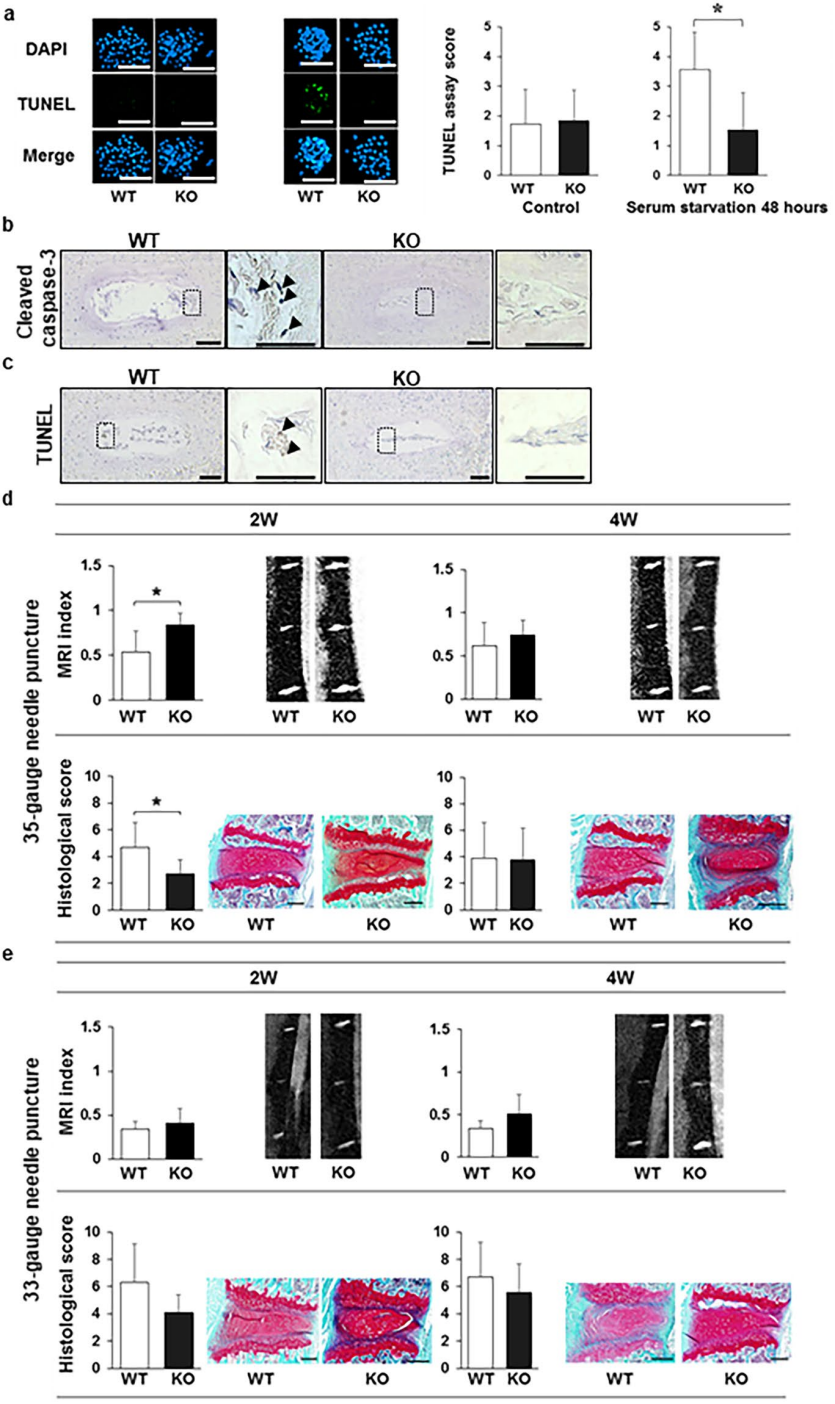
Caspase-3 knockout (KO) increases the resistance of nucleus pulposus (NP) cells to apoptosis and inhibits progression of intervertebral disc (IVD) degeneration induced by injury. (**a**) Apoptosis was evaluated using the terminal deoxynucleotidyl transferase-mediated deoxyuridine triphosphate-biotin nick end labeling (TUNEL) assay in colony-forming unit spheres (CFU-S) from wild-type (WT) and KO mice subject to the serum starvation model (*p < 0.05). CFU-S were cultured on Lab-Tek Chamber Slides (Thermo Fisher Scientific) in Eagle’s minimum essential medium (Sigma-Aldrich) supplemented with 10% fetal bovine serum (FBS; Nichirei Bioscience, Tokyo, Japan), 0.5% penicillin/streptomycin (Wako, Osaka, Japan), and 0.25 μg/ml Fungizone (Thermo Fisher Scientific). The slides were placed in a humidified incubator (maintained at 37 °C and 5% O_2_ and CO_2_). Thereafter, CFU-S were incubated for 48 h in Eagle’s minimum essential medium (Sigma-Aldrich) supplemented with 0.5% penicillin/streptomycin (Wako) and 0.25 μg/ml Fungizone (Invitrogen). The nuclei were counterstained with DAPI, and the proportions of TUNEL-positive-cells to DAPI stained cells in each CFU-S was calculated. The proportions and intensities of TUNEL-positive-cells in each CFU-S were inverted into the semi-quantitative score ranging from 0 to 5 as the TUNEL assay score. Scale bars, 200 μm. Data are the mean ± SD. (**b**,**c**) Immunohistochemistry was used to determine the cleaved caspase-3 (**b**) expression and TUNEL positivity (**c**) in the NP of punctured IVDs of WT and KO mice. Arrows indicate positive cells. Scale bars, 100 μm; 50 μm in magnified images. The representative images for cleaved caspase-3 were two weeks after 33 G needle puncture for WT and two weeks after 35 G needle puncture for KO mice. The images for TUNEL were two weeks after 33 G needle puncture for WT and two weeks after 35 G needle puncture for KO mice. (**d,e**) MRI and histological analyses were used to detect degenerative changes in IVDs punctured using a 35 gauge (G) (**d**) or 33 G (**e**) needle. The MRI index was obtained as the product of the NP area and average signal intensity, using Analyze 10.0 software (AnalyzeDirect). The histological scores were obtained by adding the AF and NP scores, each ranging from 0 to 5. A total score ranging from 0 (intact) to 10 (the severest) was used to determine the severity of degeneration. The data represent the mean ± SD. Scale bars, 200 μm.

First, we measured apoptosis *in vitro* using the terminal deoxynucleotidyl transferase-mediated deoxyuridine triphosphate-biotin nick end labeling (TUNEL) assay, which measures DNA fragmentation, following serum starvation to simulate conditions that stimulate apoptosis^7,14,30^. We compared the apoptotic cell rate in the NP cell colonies obtained from mice younger than six months (Supplementary Fig. S2a) using a serum starvation model^7,14,30^ *in vitro*. The proportion of the TUNEL-positive cells out of 4′, 6-diamidino-2-phenylindole (DAPI)-stained cells is represented by a semi-quantitative score. The scores were identical between the WT and Casp-3 KO groups in the serum-starved control. However, it was significantly lower in the Casp-3 KO group than in the WT group after 48 h of serum starvation^7,14,30^ (Fig. 4a). These results suggest that NP cells from Casp-3 KO mice had enhanced apoptosis resistance *in vitro*.

Next, cleavage of caspase-3 and TUNEL-positivity were assessed using immunohistochemistry. Cleaved caspase-3 and TUNEL-positive cells were identified in injured discs of WT, but not Casp-3 KO, mice (Fig. 4b,c). The representative images for cleaved caspase-3 were two weeks after the 33 G needle puncture for WT and two weeks after 35 G needle puncture for Casp-3 KO mice. The images for TUNEL were two weeks after the 33 G needle puncture for WT and two weeks after the 35 G needle puncture for Casp-3 KO mouse.

Finally, the effect of the Casp-3 KO in the injured IVD was assessed *in vivo*. We used our needle puncture IRD model^22^ in 11-week-old mice. We compared the degree of degeneration between 13 and 15-week-old (young) (as well as 19 and 23-week-old, only with a 33 gauge (G) needle puncture) WT and Casp-3 KO mice by magnetic resonance imaging (MRI) and histological evaluations. The MRI index was substantially higher, and the histological degenerative score was significantly lower, in the punctured IVDs of Casp-3 KO mice than in WT mice two weeks after puncture using a 35 G needle (Fig. 4d), indicating lower IVD degeneration in Casp-3 KO mice. However, the MRI index and histological degenerative scores converged over time between the two groups until the difference became non-significant four weeks after the 35 G needle puncture (Fig. 4d). The result indicates degenerative changes occurred in Casp-3 KO mice but were delayed compared to WT mice.

Since the difference in degenerative changes between WT and Casp-3 KO mice became non-significant four weeks after the needle puncture, we did not evaluate 8 or 12 weeks after the puncture to reduce the amount of animal sacrifice. In contrast, the MRI index and histological degenerative score were not significantly different in the punctured IVDs of Casp-3 KO mice compared to those in WT mice 2, 4, 8, and 12 weeks after the 33 G needle puncture (Fig. 4e, Supplementary Fig. S2b).

The present results show that NP cells in Casp-3 KO mice enhanced apoptosis resistance *in vitro* and apoptosis suppression in NP of Casp-3 KO mice *in vivo*. A highly destructive injury by the 33 G needle puncture to IVDs caused severe degeneration to WT mice, and the effect of the Casp-3 KO did not alter the degenerative changes. However, a less destructive injury by the 35 G needle puncture to IVDs caused moderate degeneration in WT mice, and Casp-3 KO decelerated the progression of degenerative changes.

### Extracellular matrix degradation enzymes are upregulated in NP cells *in vitro* and the IVDs *in vivo* with more degeneration in old Casp-3 KO mice

Aging is another cause of IVD degeneration. Therefore, we investigated the extracellular matrix degradation enzyme levels in NP cells of young (younger than 6-month-old mice) and old (older than 14-month-old mice) WT and Casp-3 KO mice *in vitro*. We also analyzed the expression levels of an extracellular matrix degradation enzyme and apoptosis using our *in vivo* age-related spontaneous IVD degeneration model^23^. MMP-3 and ADAMTS5 levels were measured as a correlate of extracellular matrix degradation, which is related to IVD degeneration. Finally, the IVD degeneration in young (6-month-old mice) and old (14- and 18-month-old mice) WT and Casp-3 KO mice were histologically compared.

**Figure 5:**
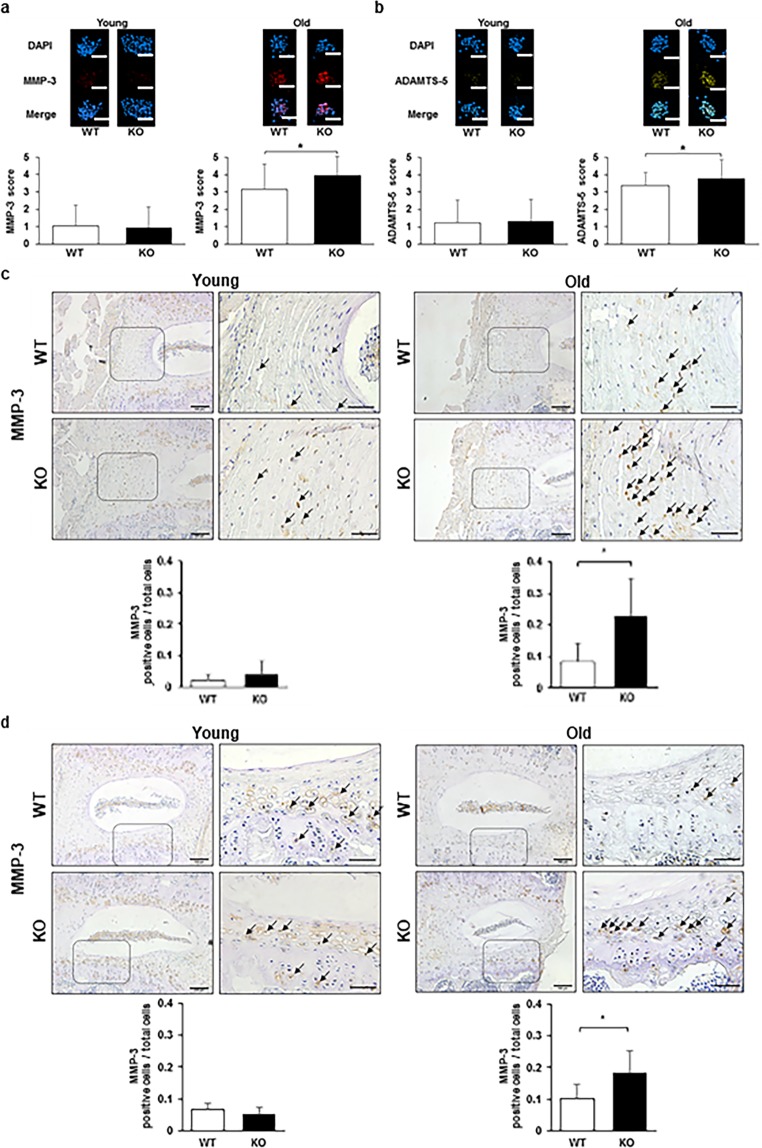
Caspase-3 KO enhances the expression of extracellular matrix degradation enzymes with age. (**a**,**b**) Matrix metalloproteinase-3 (MMP-3) and a disintegrin and metalloproteinase with thrombospondin motifs 5 (ADAMTS-5) expression levels in the CFU-S of young (younger than 6-month-old) and old (older than 14-month-old) WT and KO mice without applying any specific treatment (*p < 0.05). The nuclei were counterstained with DAPI, and the proportions of MMP-3 and ADAMTS-5-positive-cells out of DAPI stained cells in each CFU-S were calculated. The proportions and intensities of positive cells in each CFU-S were inverted into the semi-quantitative score ranging from 0 to 5 for MMP-3 and ADAMTS-5. Forty-five CFU-S replicates for MMP-3 were used for WT and KO mice in young and old groups. For ADAMTS-5, CFU-S replicates were 36 in WT mice and 44 in KO mice in the young group, as well as 34 replicates in WT mice and 45 replicates in KO mice in the old group. Every three mice were used to harvest NP for WT and KO groups in young and old groups. Scale bars, 200 μm. Data are the mean ± SD. (**c**,**d**) Immunohistochemistry was performed to determine the MMP-3 expression in the AF (**c**) and EPs (**d**) of WT and caspase-3 KO mice. Arrows indicate positive cells. Scale bars, 100 μm; 50 μm in magnified images. The proportions of positive cells out of total cells in the field were calculated. Data are the mean ± SD (*p < 0.05).

MMP-3 and ADAMTS5 expression levels were comparable in NP cell colonies grown from young WT and Casp-3 KO mice, while they were significantly higher in NP cell colonies from old Casp-3 KO mice than in those from old WT mice (Fig. 5a,b). Similarly, MMP-3-positive cell rates of the total cells were identical in the AFs and EPs of young WT and Casp-3 KO mice, and were significantly higher in old Casp-3 KO mice than old WT mice, as determined by immunohistochemical analysis (Fig. 5c,d) (notochordal cells were clustered in the center of NP, and the positive area of MMP-3 was crossing the borderline among the notochordal cells. Therefore, we thought that it was difficult to analyze the disc NP for MMP-3 positive cells). Accordingly, extracellular matrix degradation enzyme expression levels were enhanced in the IVDs cells of old Casp-3 KO mice.

**Figure 6:**
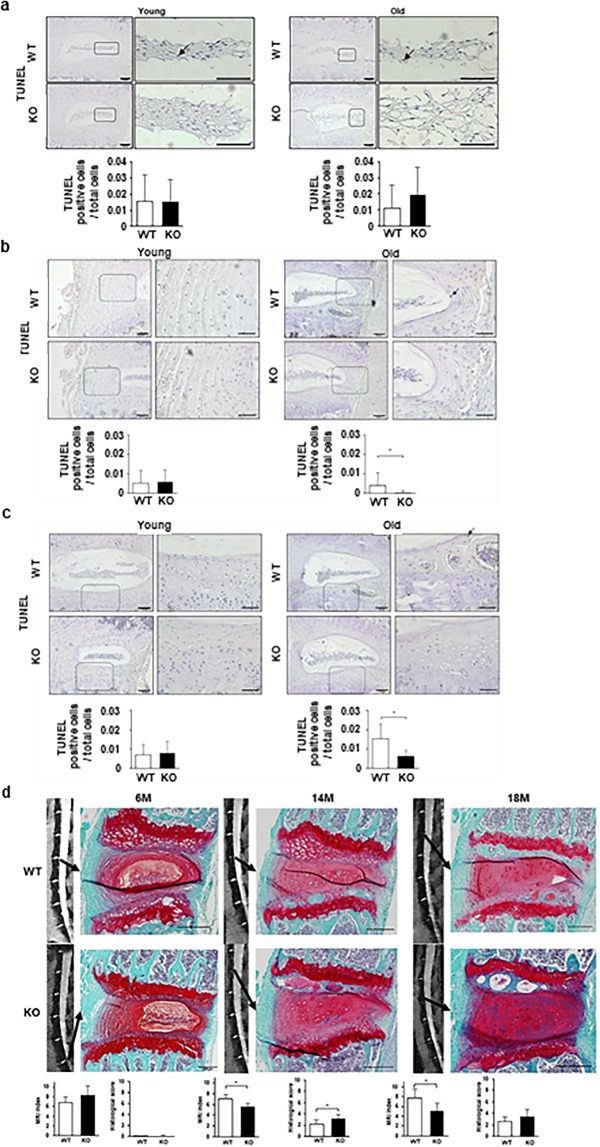
Age-related spontaneous IVD degeneration (ARD) deteriorated in caspase-3 KO mice; however, apoptotic cells did not increase. (**a**–**c**) TUNEL assay using immunohistochemistry in the NP (**a**), AF (**b**), and EP (**c**) of young (13-week-old) and old (14-month-old) WT and KO mice. Arrows indicate positive cells. Scale bars, 100 μm; 50 μm in magnified images. The proportion of positive cells to total cells in the field were calculated. Data are the mean ± SD. (**d**) MRI and histological analyses to detect degenerative changes in the IVDs of the lumbar spine in 6- to 18-month-old WT and KO mice (*p < 0.05). Scale bars, 200 μm. Data are the mean ± SD.

Apoptosis, measured as TUNEL within the total cells in the NP, was similar between WT and Casp-3 KO mice in both the young (13-week-old mice) and old (14-month-old mice) groups as determined by immunohistochemical analysis (Fig. 6a). The AF and EP rates were also equivalent between young WT and Casp-3 KO mice but were significantly lower in old Casp-3 KO mice than in old WT mice (Fig. 6b,c).

The MRI indexes and histological degenerative scores were not significantly different in six-month-old (young) WT and Casp-3 KO mice, and histological degenerative scores were almost zero in both groups of young mice (Fig. 6d). In 14-month-old (old) mice, the MRI index was significantly lower, and the histological degenerative score was significantly higher in Casp-3 KO mice than in WT mice (Fig. 6d). Increased safranin-O positivity was observed in 14-month-old Casp-3 KO mouse discs; however, the morphology of the NP matrix was more disorganized, and the number of notochordal cells was lesser in Casp-3 KO mice than that in WT mice. In 18-month-old mice, the results were similar; the MRI index was the only statistically significant difference (Fig. 6d). In contrast to the findings with the IRD model, degeneration was more severe in Casp3-KO old mice, aged 14 and 18 months, than in age-matched WT mice in the MRI and histological evaluations (Fig. 6d).

The present results suggest that Casp-3 KO deteriorated the IVD degeneration after aging, correlating positively with the expression of extracellular matrix degradation enzymes, but with no correlation with apoptosis level.

### Higher expression of senescence markers in IVDs of old Casp-3 KO mice than in old WT mice

**Figure 7:**
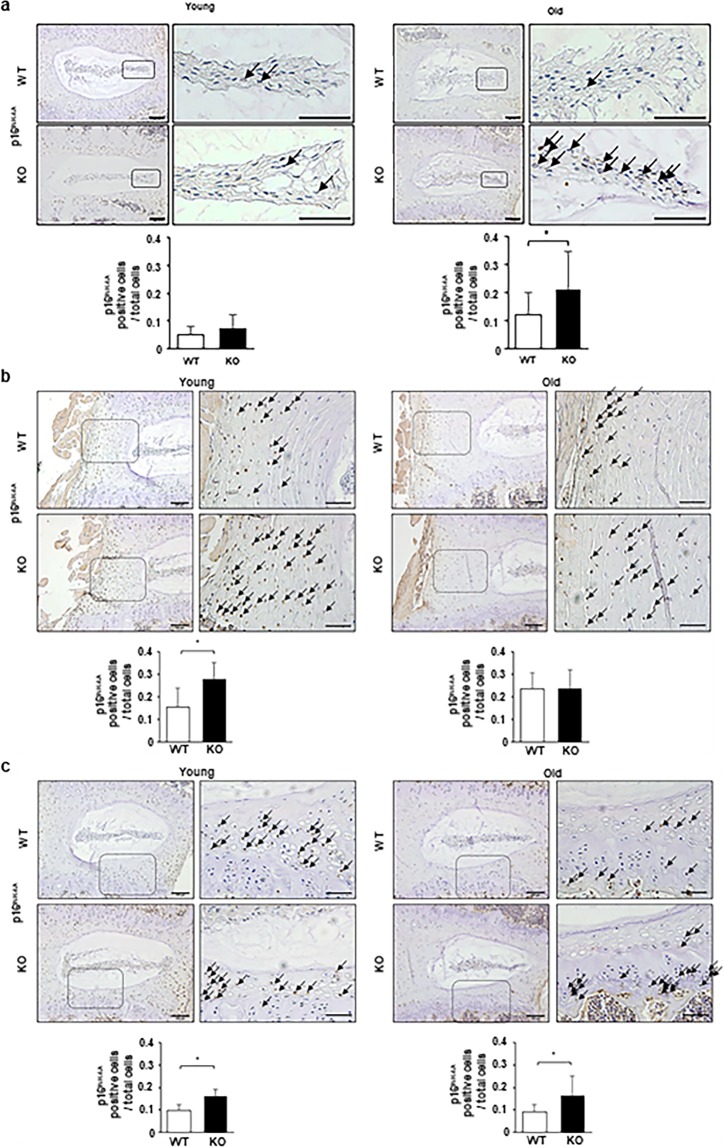
Higher expression of a cellular senescence marker is evident in IVDs of old caspase-3 KO mice than in old WT mice. (**a**–**c**) Immunohistochemistry was performed to determine p16^INK4A^ expression in the NP (**a**), AF (**b**), and EP (**c**) of young (13-week-old) and old (14-month-old) WT and KO mice. Arrows indicate positive cells. Scale bars, 100 μm; 50 μm in magnified images. The proportion of positive cells to total cells in the field were calculated. Data are the mean ± SD (*p < 0.05).

The observed discrepancies in the degenerative changes and apoptotic cell rates between the IRD and ARD models in the Casp-3 KO mice suggest different pathological mechanisms. We hypothesized that cellular senescence, but not apoptosis, may be a significant contributing factor to ARD. To test this, we counted the cells immunopositive for p16^INK4A^ (a marker of cellular senescence) in IVDs and EPs from 13-week-old (young) mice from the IRD model eliminating the punctured IVD, and 14 month old (old) mice from the ARD model. In young mice, the p16^INK4A^ positivity rates within the total cells were higher in the AFs and EPs of Casp-3 KO mice than in those of WT mice, with no difference between genotypes in the NPs (Fig. 7a–c). In old mice, the rates within the total cells were higher in the NPs and EPs of Casp-3 KO mice than WT mice, with no difference between genotypes in the AFs (Fig. 7a–c). β-galactosidase expression levels were also confirmed in the NP cell colonies of young (younger than 6-month-old) and old (older than 14-month-old) WT and KO groups by immunocytofluorescence (Supplementary Fig. S3a). The expression levels in the KO group were higher than those in the WT group, regardless of age, although the differences were not significant (Supplementary Fig. S3a).

In summary, we found cellular senescence marker expressions were higher in the IVDs of old Casp-3 KO mice compared to old WT mice, suggesting senescent cells accumulated in Casp-3 KO mice from age. Senescent cell accumulation would explain higher extracellular matrix degradation enzyme expression and deteriorated IVD degeneration in the old Casp-3 KO mice. Therefore, cellular senescence, but not apoptosis, can be a significant contributing factor to ARD.

### Casp-3 KO mice do not show tumorigenesis in the IVDs

**Figure 8:**
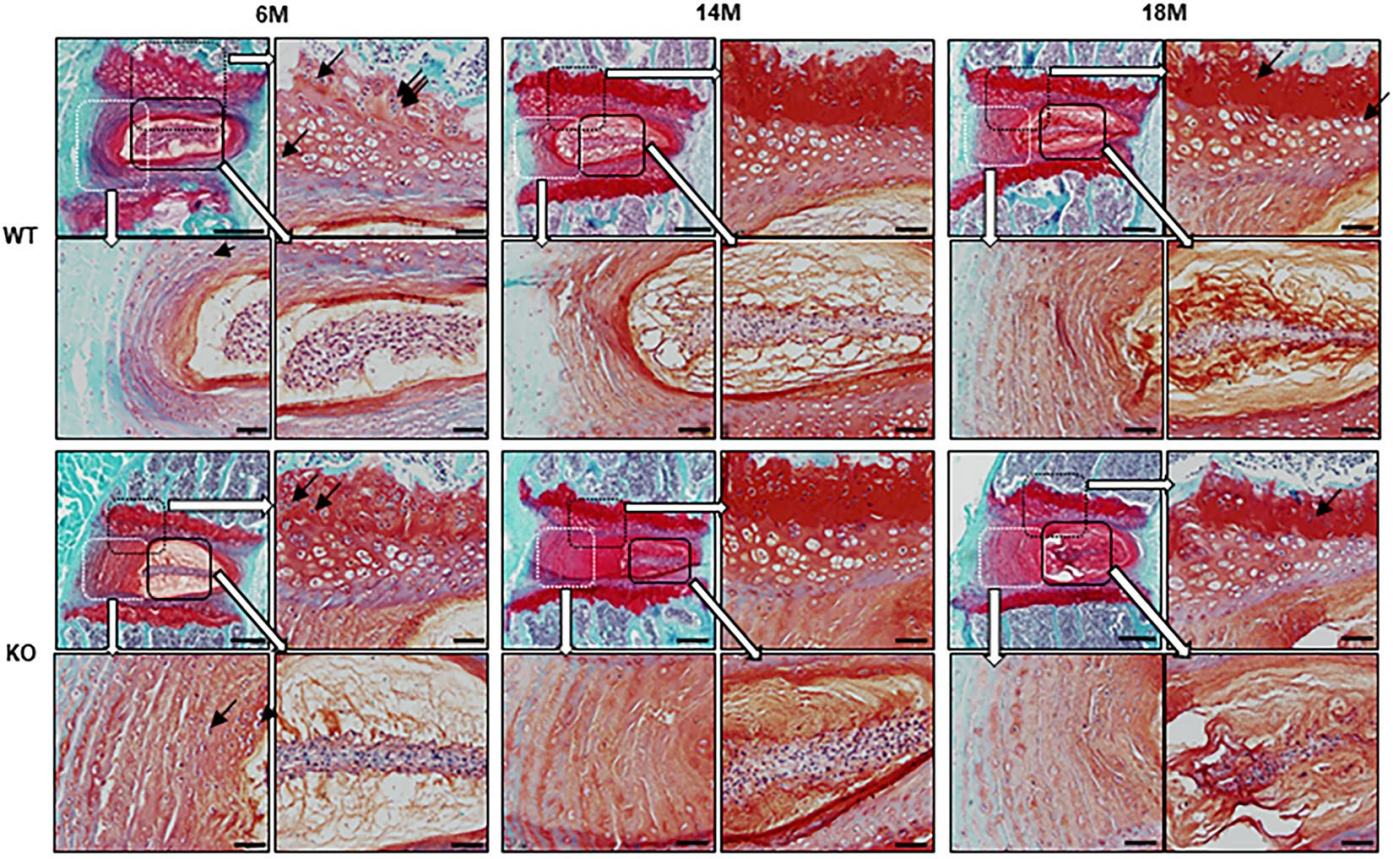
The caspase-3 KO does not cause tumorigenesis in IVDs regardless of age. Histological specimens of the IVDs stained with safranin O and fast green of WT and KO mice aged six months (6 M), 14 months (14 M), and 18 months (18 M). The black arrows indicate binucleate cells. Scale bars in the upper left image in each age of WT and KO mice, 200 μm. The upper right, lower left, and lower right images in each age of WT and KO mice correspond to the magnifications of the enclosed areas; scale bars, 50 μm.

Finally, we evaluated whether long-term caspase-3 deficiency causes tumorigenesis. To this end, we assessed the number of binucleate cells, the indicator of cell division, and tissue proliferation potential, in the IVDs of young (6-month-old mice) and old (14- and 18-month-old mice) Casp-3 KO and WT mice. Our results showed more binucleate cells in WT mice than Casp-3 KO mice, especially in the EPs of six-month-old (young) mice (Fig. 8). Few binucleate cells were observed in the IVDs of Casp-3 KO mice, suggesting that the IVDs of these mice were not highly proliferative and did not show tumorigenesis.

## Discussion

In the present study, we investigated whether the different pathological mechanisms in IRD and ARD affect the response to caspase-3 deletion and whether long-term caspase-3 inhibition results in tumorigenesis. IRD has been described in previous studies that showed that increased gene expression for type I and II collagen, fibronectin, several metalloproteinases (MMP-1, -9, and -13), various growth factors (fibroblast growth factor and transforming growth factor-alpha), pro-inflammatory cytokines (tumor necrosis factor-alpha and IL-1 beta)^19,20^, and the coexpression of FasL with Fas induce the apoptosis of disc cells^21^. ARD has also been described wherein the accumulation of senescent cells and their altered pattern of gene expression implicates cellular senescence^31,32^, and there is a correlation between the expression of senescent biomarkers and increased gene expression of the degrading enzymes MMP-13 and aggrecanase (ADAMTS-5)^26^. However, IRD and ARD have not been previously compared based on their etiology. The present study is the first to compare the pathological differences between IVD degeneration related to injury and aging (Supplementary Fig. S3b). Casp-3 KO mice showed enhanced apoptosis resistance *in vitro* and delayed progression of injury-induced degeneration *in vivo*, consistent with studies indicating apoptosis’ role in IVD degeneration^7,12,14,15,18,33^.

High cellular senescence marker levels in Casp-3 KO mice indicate accumulated senescent cells in IVDs, especially in old mice. Senescent cells accumulation and elevated extracellular matrix degradation enzymes corresponded to an enhanced, senescence-associated, secretory phenotype (SASP)^34^ in old Casp-3 KO mice. SASP is a senescent cell phenotype that secretes increased proteins associated with inflammation and tumorigenesis that is related to age-related pathologies. It was documented as one of the IVD degeneration pathologies since elevated expression of extracellular matrix degradation enzymes like MMP-3 and ADAMTS-5 accompanied elevated p16^INK4A^ expression^24^. Our previous study can also explain our outcome. Western blot analysis using rat NP cells demonstrated that p21^Cip1^ protein expression increased after 6 h and decreased after 48 h of serum starvation^30^. In addition, p53 protein expression increased after 6 and 48 h of serum starvation^30^. In contrast, p15^Ink4b^ and p16^Ink4a^ protein expression remained unchanged even after 48 h of serum starvation^30^. Furthermore, although p21^Cip1^ protein expression decreased after 48 h of serum starvation, p21^Cip1^ protein expression was maintained in caspase-3 siRNA-transfected cells^30^. These results can partially explain the effect of caspase-3 suppression that enhances cellular senescence. In addition, cell densities were higher in the NPs and AFs of old Casp-3 KO mice than old WT mice, but no tumorigenesis was observed. Although tumorigenesis cannot be limited only to the assessment of the intervertebral discs, previous studies have shown the phenotype of other organs in Casp-3 KO mice and revealed that tumorigenesis did not exist in the other organs^35–37^. We investigated the IVDs in Casp-3 KO mice because they were not evaluated in previous studies. In addition, 18-month-old Casp-3 KO mice were the oldest mice we could evaluate because many Casp-3 KO mice died before 18 months of age.

We previously suggested temporarily inhibiting the caspase-3 gene by injection as an anti-apoptotic therapy for IVD degeneration^7,12^. We defined the indication for this treatment as IRD but not ARD from the findings in the present study. IRD progression can be delayed by temporarily inhibiting caspase-3 to enhance the resistance of IVD cells to apoptosis, without a high risk of tumorigenesis. However, with constitutive caspase-3 inhibition, as shown in the present study, there is a risk of ARD deterioration because of accumulated senescent cells and subsequently enhanced SASP.

As we have defined the indication of anti-apoptotic treatment for IVD degeneration as IRD, but not ARD, we suggest that the present anti-apoptotic strategy could be applied to spinal injuries with damaged IVDs.

In addition, our previous study showed that caspase-3 silencing inhibited biomechanical overload-induced IVD degeneration^12^. A biomechanical overload on the IVDs can be classed as a traumatic force and can be included in IRD. An example of this in the clinical setting may be adjacent segment disease^3^ after spinal fusion surgery. According to Yurube *et al*.^38^, MMP-3 upregulation level is more predominant than that of ADAMTS-5, and the level of ADAMTS-5 decreased in the later stage of degeneration during mechanical loading. The level of expression was higher in NP compared with that in AF. However, there is lack of evidence regarding the expression patterns of the catabolic enzymes in IVD degeneration by injury and aging. We plan to work on this issue in the future.

This study has a few limitations. First, molecular quantitative methods such as western blotting could not be performed because only a small amount of tissues and cells was obtained from the IVDs of mice. Second, the group numbers at each sacrifice time and the number of male and female mice in each group were different. Although Casp-3 KO mice were difficult to obtain, the minimum number (n = 8) was set based on previous studies^7,12,39,40^. Meanwhile, the group numbers of WT mice were set based on our previous study regarding injury-related IVD degeneration model using WT mice^22^. The variation of the group numbers was considered in statistical analyses. As for gender distributions, equal numbers of male and female mice were difficult to obtain during the experimental period. Therefore, the possible effects that might be caused by sex difference are still unknown. Third, some assessments were performed *in vitro* vs histologically. The assessments for molecular pathology were mainly performed using immunocytofluorescence or immunohistochemistry. We selected the measure of better reactivity in antibodies for some assessments. Fourth, weak signals cannot be effectively detected by immunochemistry; therefore, immunofluorescent detection should be performed in future. There is a possibility of adjacent disc effect using the IRD model eliminating the punctured IVD (L1/2, L2/3, L3/4, L5/6, and L6/S discs) for evaluating p16^INK4A^ positivity, although in previous studies, adjacent discs have been used as control groups^41^ or the sequential levels of discs have been punctured to examine the effects of the therapy^42^. Still, the present study provides further evidence that anti-apoptotic treatment of IVD degeneration represents a new avenue of investigation for treating spinal trauma.

## Methods

### Mouse breeding

All animal procedures in this study were approved by the Institutional Animal Care and Use Committee of Hokkaido University. All procedures were carried out in accordance with the approved guidelines. Inbred C57BL/6 WT mice were obtained from Sankyo Labo Service Corporation (Tokyo, Japan), and two male Casp-3 KO mice with a C57BL/6 background, aged 5 and 8.5 weeks, were obtained from the Jackson Laboratory (Bar Harbor, ME, USA). Breeding and husbandry were performed as previously described^22,23^. The mice were bred and housed under specific pathogen-free conditions at Hokkaido University Creative Research Institution Platform for Research on Biofunctional Molecules^22,23^. They were kept in cages at room temperature (23 °C ± 2 °C) and humidity of 50% ± 10% under standard laboratory conditions with a 12 h light/dark cycle^22,23^. They were allowed unrestricted cage activity and *ad libitum* access to food and water. A standard laboratory diet, Labo MR Stock (Nosan Corporation, Yokohama, Japan) and sterilized tap water were provided as sources of food and water^22,23^. The number of Casp-3 KO mice was increased through breeding with WT mice, followed by breeding borne heterozygotes with heterozygotes or Casp-3 KO mice. We defined mice aged six months or younger as young, and those aged 14 months or old as old, based on our previous study^23^. Mild but significant IVD degeneration was observed in WT mice aged 14 months.

### Mouse genotyping

For genotyping, the tip of the tail was cut and incubated in 50 µl Tris-acetate-ethylenediamine-tetraacetic acid buffer (Kanto Chemical Company Incorporated, Tokyo, Japan) at 95 °C for 5 min, then incubated in 1.5 µl Proteinase K from *Tritirachium* (Sigma-Aldrich, St. Louis, MO, USA) at 60 °C for 60 min and 100 °C for 5–10 min. Casp-3 KO mice were genotyped by standard polymerase chain reaction (PCR) using 1 µl tail lysate, Takara Ex Taq (Takara Bio, Shiga, Japan), 10X Ex Taq Buffer (Takara Bio), and dNTP Mixture (Takara Bio), according to the manufacturer’s instructions. Primers for Casp-3 KO mice (sequences: 5′-GCG AGT GAG AAT GTG CAT AAA TTC-3′, 5′-GGG AAA CCA ACA GTA GTC AGT CCT-3′, and 5′-TGC TAA AGC GCA TGC TCC AGA CTG-3′) were synthesized by Takara Bio and diluted in MilliQ water to 0.2 µM. For the PCR, a thermal cycler (Takara Bio) was programmed as follows: (1) 94 °C for 2 min; (2) 94 °C for 20 sec; (3) 65 °C for 15 sec, decrease by 0.5 °C per cycle; (4) 68 °C for 10 sec; (5) repeat steps 2–4 for 10 cycles; (6) 94 °C for 15 sec; (7) 60 °C for 15 sec; (8) 72 °C for 10 sec; (9) repeat steps 6–8 for 28 cycles; (10) 72 °C for 2 min; (11) 10 °C hold.

### Whole-body skeletal staining

A newborn WT mouse and Casp-3 KO mouse were euthanized with an intraperitoneal injection of 0.5 mg pentobarbital sodium, and they were fixed with 70% ethanol for 5–7 days, followed by resection of soft tissues. Dehydration and delipidation were performed using 99.5% ethanol. The cartilage was stained with 0.05% Alcian blue, before dehydration and decolorization using 99.5% ethanol. The remaining soft tissue was dissolved, and transparency was obtained using a 2% potassium hydroxide solution. The calcified bone was stained with 0.005% Alizarin red, followed by decolorization using a 1% potassium hydroxide/20% glycerin mixed liquor.

### Analysis of growth using body weight

Two sets of littermates (one male and four female WT mice and two male Casp-3 KO mice), born from the same couple of heterozygotes, were analyzed for changes in body weight from three to 14 weeks after birth. In addition, WT and Casp-3 KO body weights were compared at 14 months old (eight male and three female WT mice; four male and six female Casp-3 KO mice) and 18 months old (seven male and four female WT mice; eight male and three female Casp-3 KO mice).

### Injury-related IVD degeneration model

We used our *in vivo* model to elicit IRD^22^ in 11-week-old WT and Casp-3 KO mice. Surgery was performed to puncture mice in the central or dorsal regions using a 35 or 33 G needle. We euthanized 22 WT and 16 Casp-3 KO mice with an intraperitoneal injection of 5 mg pentobarbital sodium, two or four weeks after puncturing with a 35 G needle (2 weeks: WT: female, 9; male, 4; Casp-3 KO: female, 3; male, 5; 4 weeks: WT: female, 4; male, 5; Casp-3 KO: female, 3; male, 5). After puncturing with a 33 G needle, 39 WT and 40 Casp-3 KO mice were euthanized 2, 4, 8, or 12 weeks for further analysis (2 weeks: WT: female, none; male, 10; Casp-3 KO: female, 8; male, 2; 4 weeks: WT: female, 6; male, 4; Casp-3 KO: female, 2; male, 8; 8 weeks: WT: female, 5; male, 5; Casp-3 KO: female, 6; male, 4; 12 weeks: WT: female, 3; male, 6; Casp-3 KO: female, 5; male, 5) (Supplementary Fig. S4). L3/4, L4/5, and L5/6 IVDs were harvested for evaluation.

### Age-related spontaneous IVD degeneration model

We used our *in vivo* model to elicit ARD^23^ in 32 WT and 32 Casp-3 KO mice aged 6, 14, and 18 months. Again, we defined mice aged 6 months or younger as young and those aged 14 months or older as old on the basis of our previous study^23^. The number of mice from each sex in each age group were as follows; 6 months: WT: female, 4; male, 6; Casp-3 KO: female, 5; male, 5; 14 months: WT: female, 2; male, 9; Casp-3 KO: female, 7; male, 4; 18 months: WT: female, 4; male, 7; Casp-3 KO: female, 3; male, 8 (Supplementary Fig. S5). They were euthanized with an intraperitoneal injection of 5 mg pentobarbital sodium, and all lumbar IVDs (L1/2, L2/3, L3/4, L4/5, L5/6, and L6/S) were harvested for evaluation.

### MRI and evaluation

Mid-sagittal images of the lumbar IVDs were analyzed using MRI to document degenerative changes^7,12,22,23,43^. More precisely, T2-weighted mid-sagittal images of the discs were analyzed using a 7.0-T MR scanner (Varian Unity Inova; Varian Medical Systems, Palo Alto, CA, USA)^7,12,22,23,43^. The sagittal image slices were analyzed quantitatively using Analyze 10.0 software (AnalyzeDirect, Overland Park, KS, USA), as previously reported^7,12,22,23,43^.

We used the MRI index (the product of the NP area and average signal intensity) to quantify the alterations in the NP^7,12,22,23,43^. Normalization for signal intensity variation was performed against the average value of non-punctured L3/4 and L5/6 IVDs for the IRD models^22^ and the signal intensity of the L6 vertebral or sacral bone for the ARD models^23^. Data are expressed as ratios of the results obtained when using each model’s normalization reference^22,23^. All image assessments were performed by two independent observers blinded to the experimental grouping. The quantitative data are presented as the mean of three quantifications and were used to compare WT and Casp-3 KO mice.

### Histology

Each IVD was fixed in 10% neutral buffered formalin solution for 48 h, followed by decalcification with 10% EDTA for 2–4 weeks, then embedded in paraffin. Mid-sagittal sections were obtained and stained with safranin O and fast green^7,22,23^. Our previously proposed classifications for the IRD^22^ and ARD models^23^ were used to evaluate degeneration. For each classification type, the maximum points represent the severest degeneration^22,23^. The degeneration was evaluated for punctured IVDs with the IRD model^22^ (35 G, 22 WT, and 16 Casp-3 KO mice; 33 G, 39 WT, and 40 Casp-3 KO mice) and all the lumbar IVDs (L1/2, L2/3, L3/4, L4/5, L5/6, and L6/S) in the ARD models^23^ (32 WT and 32 Casp-3 KO mice). The regions of interest in the figures for counting cells and measuring tissue areas were selected by focusing the tissue center to the center of the figure. Cells were counted manually using a cell counter (each six WT and Casp-3 KO mouse for young and old mice). Tissue areas were measured using the Area Measurement tool in ImageJ, version 1.47 (National Institutes of Health, Bethesda, MD, USA; each of the six WT and Casp-3 KO mice for young and old mice). NP: the definition of cell area and total area are shown in Fig. 1a. The quantification of the total cell number, cell area, cell area density, total area, and cell density in the total area was performed. Both 27 IVDs from six mice were evaluated for young WT and Casp-3 KO mice, respectively. Overall, 35 IVDs from six old WT mice and 36 IVDs from six old Casp-3 KO mice were assessed. AF, EP: quantification of the total cell number, area, and cell density was performed. For AF, 27 IVDs from six young WT mice, 30 IVDs from six young Casp-3 KO mice, and 36 IVDs from six old WT and Casp-3 KO mice were evaluated. For EP, 27 IVDs from six young WT mice, 30 IVDs from six young Casp-3 KO mice, and 36 IVDs from six old WT and Casp-3 KO mice were evaluated. Two independent blinded observers performed all histological assessments, and the quantitative data are presented as the mean of three quantifications for comparison between WT and Casp-3 KO mice.

### Immunohistochemistry

#### MMP-3, p16^INK4A^, and cleaved caspase-3

Immunohistochemical slides for MMP-3, p16^INK4A^ and cleaved caspase-3 were prepared as follows. The paraffin sections were deparaffinized and washed in running water. Antigen retrieval was performed and slides were washed with water. They were treated with 3% hydrogen peroxide solution for 5 min, followed by three 5 min treatments with Tris-buffered saline (TBS). The sections were incubated with the primary antibody for 60 min and rinsed three times with TBS for 5 min each time. The sections were then exposed to a peroxidase kit (EnVision System; Dako, Santa Clara, CA, USA) for 30 min, followed by three washes with TBS for 5 min, and the color was developed with 3,3′-diaminobenzidine hydrochloride (Dako). The samples were washed with distilled water, and hematoxylin was used for nuclear counterstaining. For negative control studies, the primary antibody was replaced with normal serum from its host species. The following agents were used: MMP-3 Polyclonal Antibody (1:100; 17873-1-AP; Proteintech, Rosemont, IL, USA), EnVision + Single Reagent K4002 (Dako), Anti-CDKN2A/p16 INK4a antibody (2D9A12) (ab54210, Abcam, Cambridgeshire, UK), EnVision + Single Reagent K4000 (Dako), Cleaved Caspase-3 (Asp175) antibody (9961, Cell Signaling Technology, MA, USA), and EnVision + Single Reagent K4002 (Dako).

#### TUNEL

Immunohistochemical slides for TUNEL were similarly obtained using the TACS 2 TdT-DAB *In Situ* Apoptosis Detection Kit 4810-30-K (Trevigen, Gaithersburg, MD, USA). The regions of interest in the figures for counting the cells were selected by locating the tissue center to the center of the figure. Positive cells were counted and calculated as a ratio of the total number of cells in the entire section, allowing comparison between WT and Casp-3 KO mice. All experiments were performed on 15–18 discs from each group (each of the six WT and Casp-3 KO mice for young and old mice). All immunohistochemical assessments were conducted by two independent observers blinded to experimental groupings, and the quantitative data are presented as the mean of three evaluations^22,23^.

### Preparation of nucleus pulposus spheres

We defined mice younger than six months of age as young, and 14–18 months old as old, based on our previous study. Mild but significant IVD degeneration was observed in 14-month-old WT mice^23^. When the target age was reached, mice were euthanized by cervical dislocation. NP tissues were harvested under a microscope from the IVDs of WT and Casp-3 KO mice (n = 7–21 discs from one to three mice in each group) for *in vitro* evaluation. The tissue was digested for 10 min using TrypLE Express Enzyme (1X) (Thermo Fisher Scientific, Waltham, MA, USA) and cultured on Lab-Tek Chamber Slides (Thermo Fisher Scientific) in Eagle’s minimum essential medium (Sigma-Aldrich) supplemented with 10% fetal bovine serum (FBS; Nichirei Bioscience, Tokyo, Japan), 0.5% penicillin/streptomycin (Wako, Osaka, Japan), and 0.25 μg/ml Fungizone (Thermo Fisher Scientific). The slides were placed in a humidified incubator (maintained at 37 °C and 5% O_2_ and CO_2_) for seven days so NP spheres [colony-forming units-spherical (CFU-S^31^)] could form.

### Immunocytofluorescence

#### Type II collagen and aggrecan

The NP phenotype of CFU-S^40^ from WT mice was confirmed double-positive for type II collagen and aggrecan by immunocytofluorescence (Supplementary Fig. S2a). Hereafter, the NP phenotype of the cells was judged with CFU-S, in both WT and Casp-3 KO mice. The slides were fixed with 4% paraformaldehyde (PFA) for 48 h followed by 1% PFA for 20 min at room temperature. The samples were washed with phosphate buffered saline (PBS) for 5 min, three times, and permeabilized with PBS containing 0.1% Triton X-100 for 30 min. Next, the slides were blocked with 1% bovine serum albumin containing PBS for 60 min at room temperature, then incubated with primary antibodies against type II collagen (1:400; ab34712; Abcam) and aggrecan (1:100; ab36861; Abcam) at 4 °C. They were rinsed with PBS for 5 min, three times, and incubated with anti-rabbit IgG conjugated with Alexa 488 (1:200; A-21206; Thermo Fisher Scientific) and anti-rabbit PE (1:200; 711-116-152; Jackson ImmunoResearch Laboratories, Baltimore Pike, PA, USA) for 60 min. After the samples were washed with PBS and distilled water, the nuclei were counterstained with DAPI. The CFU-S were imaged using confocal laser microscopy (Olympus Fluoview FV300, Tokyo, Japan).

#### MMP-3 and ADAMTS-5

The CFU-S isolated from the young (younger than 6-month-old) and old (older than 14-month-old) WT and Casp-3 KO mice were treated as above and without applying any specific treatment, they were incubated with primary antibodies against MMP-3 (MMP-3; 1:50; sc-31071; Santa Cruz Biotechnology, Dallas, TX, USA) for 60 min, then with anti-goat Alexa Fluor 568 (1:200; A11057; Thermo Fisher Scientific) for 30 min. For ADAMTS-5, the slides were fixed and permeabilized similarly, followed by incubation with 10% normal blocking serum in PBS for 20 min. Next, they were incubated with primary antibodies against ADAMTS-5 (1:50; sc-83186; Santa Cruz Biotechnologies) for 60 min, then rinsed with PBS for 5 min, three times. The samples were incubated with anti-rabbit fluorescein isothiocyanate (FITC) (1:100; 111-095-003; Jackson ImmunoResearch Laboratories) for 30 min and washed with PBS and distilled water. The nuclei were counterstained with DAPI.

#### β-galactosidase

For β-galactosidase, the slides were fixed similarly followed by permeabilization with PBS containing 0.1% triton X-100 for 10 min. They were rinsed thrice with PBS for 5 min. Thereafter, they were blocked with 1% BSA containing PBS for 30 min, followed by incubation with primary antibodies against β-gal (1:500; Abcam, ab9361, Cambridgeshire, UK) for 60 min. They were rinsed thrice with PBS for 5 min and incubated with anti-chicken FITC (1:200; Jackson ImmunoResearch Laboratories) for 60 min. They were then washed with PBS and distilled water, and the nuclei were counterstained with DAPI.

### Serum starvation model

Serum starvation was performed for 48 h in the WT and Casp-3 KO CFU-S of the young (younger than 6-month-old) animals to induce apoptosis as previously described^1^. Briefly, CFU-S were incubated for 48 h in Eagle’s minimum essential medium (Sigma-Aldrich) supplemented with 0.5% penicillin/streptomycin (Wako) and 0.25 μg/ml Fungizone (Invitrogen).

### Apoptosis assay

Slides of CFU-S were subjected to TUNEL assays. After incubation, the slides were fixed with 4% PFA for 48 h, washed with PBS, and treated with 0.3% hydrogen peroxide methanol for 30 min. The slides were treated with permeabilization buffer (*In Situ* Apoptosis Detection Kit; Takara Bio) for 5 min on ice and incubated in 37 °C FITC-conjugated TUNEL stain for 60 min. Finally, nuclei were counterstained with DAPI.

The slides were imaged under a confocal laser-scanning microscope (Olympus Fluoview FV300). A semi-quantitative scoring system was used to evaluate the expression in each CFU-S, according to a previous study^44^. The staining intensity was interpreted and scored on a semi-quantitative, subjective scale as follows: none, 0; weak, 1; moderate, 2; strong, 3^44^. The immunocytofluorescence results were evaluated considering the overall proportion of positive cells: no staining, 0; 1%–50% of cells with positive staining, 1; >50% of cells with positive staining, 2^44^. In addition, for each case, the label’s intensity and frequency were then added to produce a semi-quantitative final score^44^. This scoring system takes into consideration the staining intensity (scored on a scale of 0–3) and the proportion of positive cells (scored on a scale of 0–2)^44^. The intensity and proportions of positive cells were then added to produce final scores of 0 or 2–5^44^. The number of replicates of the CFU-S was 15 in WT and 12 in KO mice in the control group; 16 in WT and 15 in KO mice in the serum starvation group. Every three mice were used to harvest NP for WT and KO groups in the control and serum starvation groups.

### Statistics

Sample sizes were based on pilot experiments or previously published studies^7,12,39,40^. For each experiment, replicates are as noted in the relevant section of the methods. No samples were excluded from the analyses. All analyses of differences between groups were performed using the Wilcoxon signed-rank test. Data are presented as the mean ± standard deviation where *p* < 0.05 is considered to be statistically significant. Statistical analyses were performed using JMP Pro (SAS Institute, Cary, NC, USA).

## Supplementary information

Supplementary Figures

## Data Availability

The datasets generated and/or analyzed during the present study are available from the corresponding author on reasonable request.
