# Supplementary material for: Caspase-3 knockout inhibits intervertebral disc degeneration related to injury but accelerates degeneration related to aging
Source: Sci Rep. 2019 Dec 18;9:19324. doi: 10.1038/s41598-019-55709-3 (PMC6920379; doi:10.1038/s41598-019-55709-3)
Supplement: Supplementary file 1 — Supplementary Figures [file 41598_2019_55709_MOESM1_ESM.pdf]

Caspase-3 knockout inhibits intervertebral disc degeneration related to injury but accelerates degeneration related to aging

Takashi Ohnishi[1], Katsuhisa Yamada[1,2], Koji Iwasaki[1], Takeru Tsujimoto[1], Hideaki Higashi[3], Taichi Kimura[4], Norimasa Iwasaki[1], and Hideki Sudo[1,2] *

[1] Department of Orthopedic Surgery, Faculty of Medicine and Graduate of Medicine, Hokkaido University, Sapporo, Hokkaido, Japan

[2] Department of Advanced Medicine for Spine and Spinal Cord Disorders, Faculty of Medicine and Graduate of Medicine, Hokkaido University, Sapporo, Hokkaido, Japan

[3] Research Center for Zoonosis Control, Hokkaido University, Sapporo, Hokkaido, Japan

[4] Department of Pathological Diagnosis, Hokkaido Medical Center, Sapporo, Hokkaido, Japan

***Corresponding Author:** Hideki Sudo, MD, PhD

Department of Advanced Medicine for Spine and Spinal Cord Disorders

Faculty of Medicine and Graduate of Medicine, Hokkaido University

N15W7, Sapporo, Hokkaido 060-8638, Japan

Tel: 81-11-706-5934,

Fax: 81-11-706-6054

E-mail: hidekisudo@yahoo.co.jp

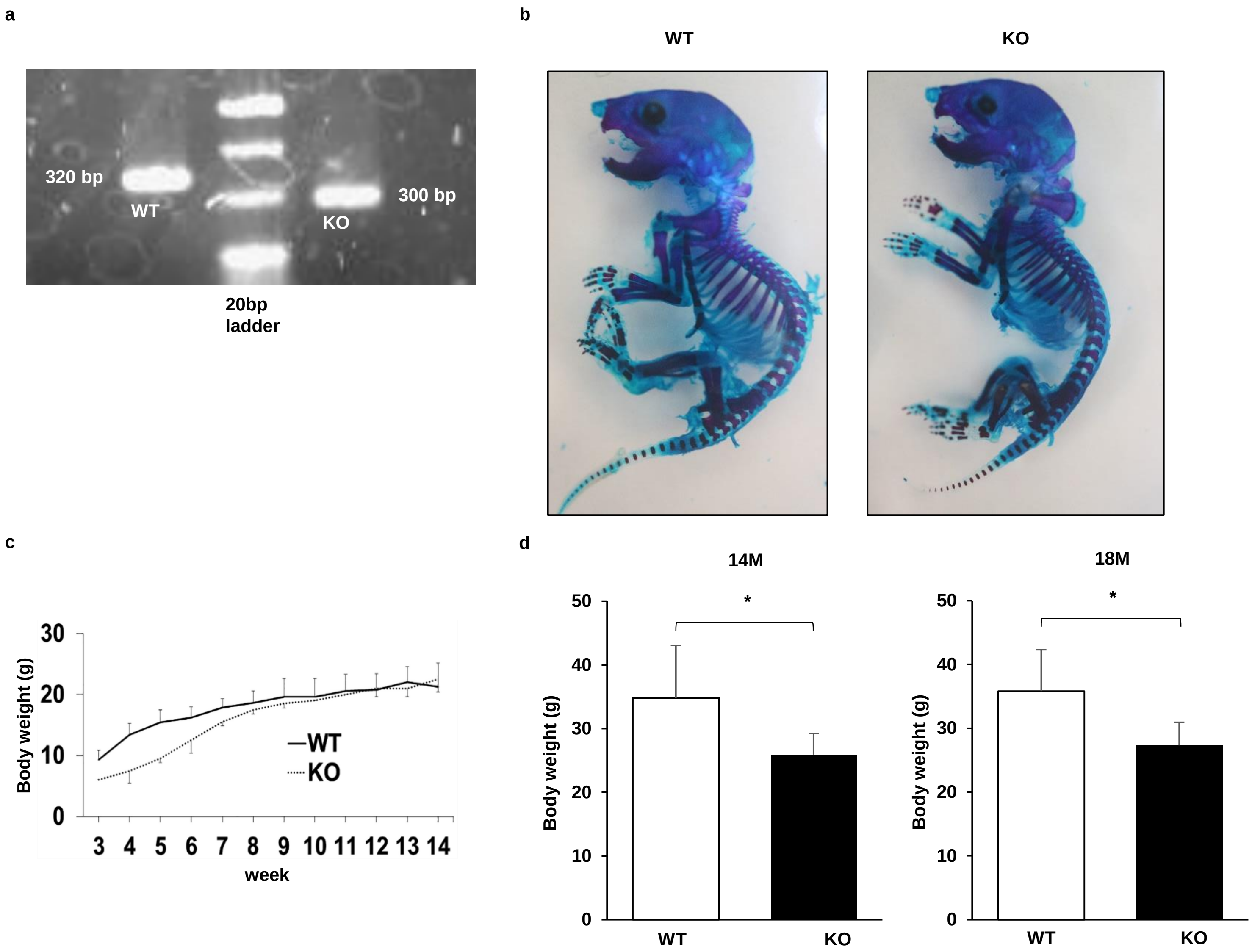

Supplementary Figure S1 Caspase-3 knockout (KO) mice have smaller body weights than wild-type (WT) mice but have no skeletal abnormality. (a) Genotyping was performed by PCR to identify KO mice. (b) Analysis of the whole-body skeletal phenotypes using double staining with Alizarin red and Alcian blue of newborn WT and KO mice. (c) Postnatal growth kinetics (body weight) of WT (n = 5, ♂1♀4) and KO (n = 2, ♂2) mice. (d) Body weight of 14-month-old (14 M) and 18-month-old (18 M) WT (14M: n=11, ♂8♀3; 18M: n=11, ♂7♀4) and KO (14M: n=11, ♂4♀7; 18M: n=11, ♂8♀3) mice. Data are the mean ± SD (*$p < 0.05$).

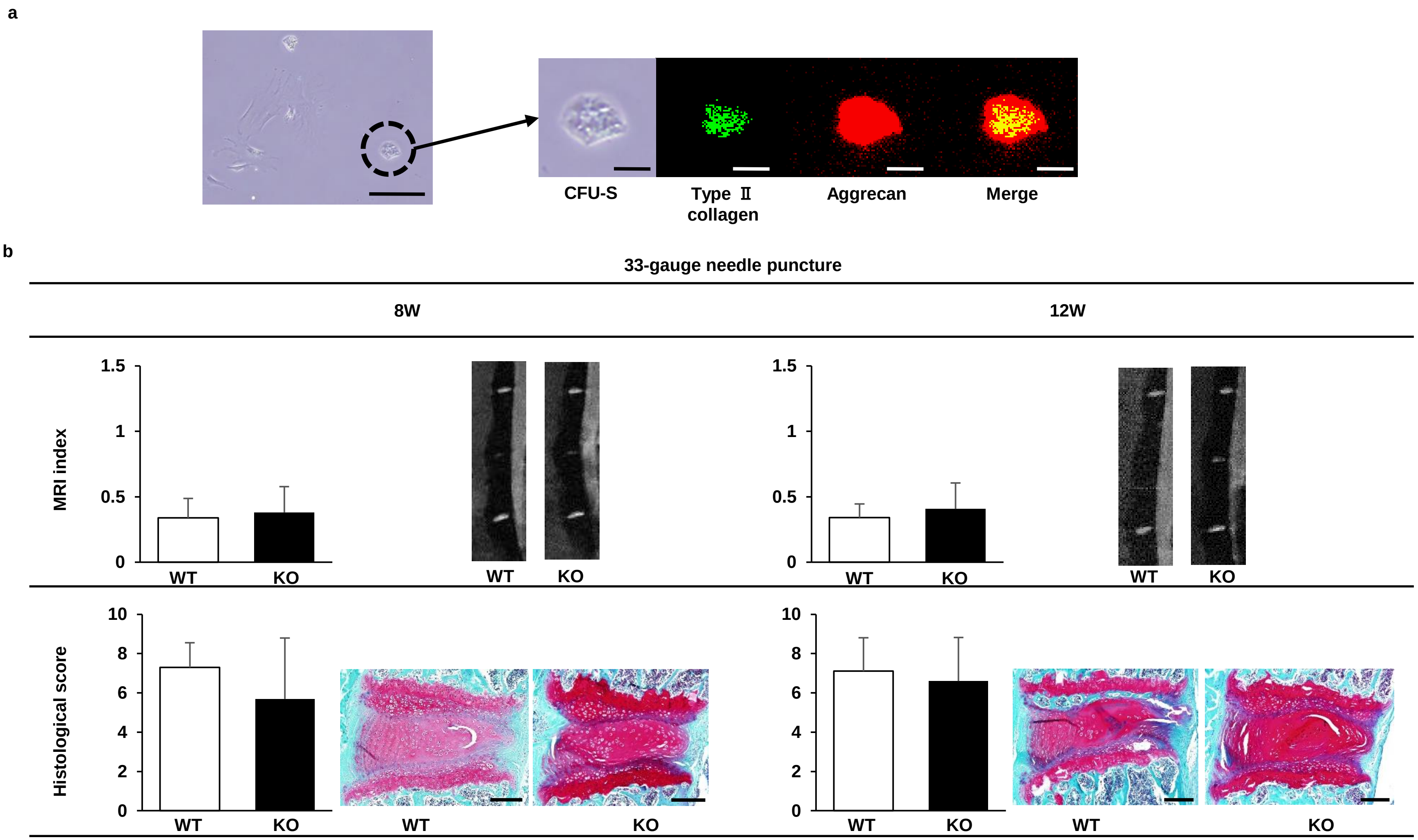

Supplementary Figure S2 Confirmation of colony-forming-unit-spheres (CFU-S) having the nucleus pulposus (NP) cell phenotype, and evidence of no significant difference in severity of IVD degeneration between WT and caspase-3 KO mice, 8 and 12 weeks after a 33 gauge (G) needle puncture. (a) Optical microscopy images of cultured NP cells in WT mice. Scale bar, 2.5 mm and 500 μm in the magnified image and immunocytofluorescence images. The phenotype of NP was confirmed by double-positivity of type II collagen and aggrecan. (b) MRI and histological analyses to detect degenerative changes in the IVDs 8–12 weeks after a 33 G needle puncture in WT and KO mice. Scale bars, 200 μm. Data are the mean ± SD.

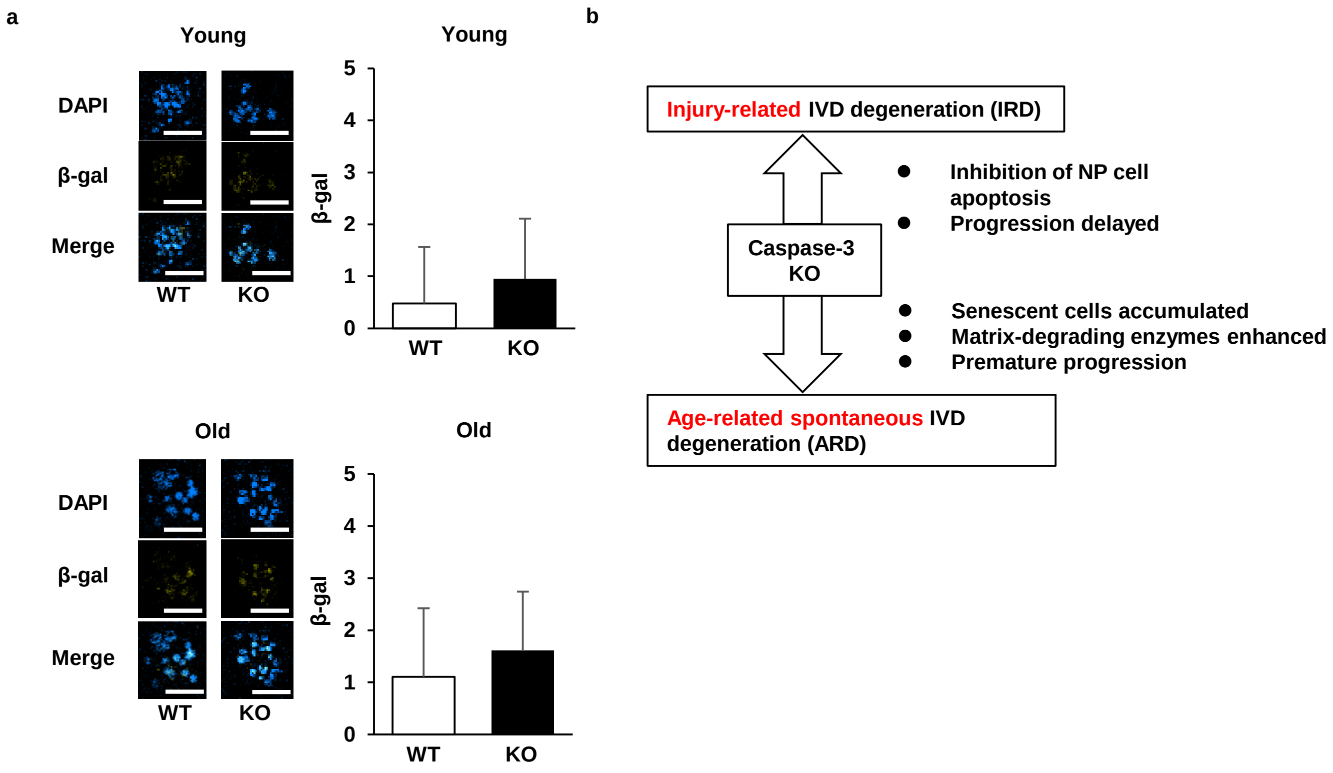

Supplementary Figure S3 Higer β-galactosidase (β-gal) expression in nucleus pulposus (NP) cell colonies of caspase-3 knockout (KO) mouse compared with wild-type (WT) mouse and pathological differences between injury-related IVD degeneration (IRD) and ARD. (a) β-gal expression levels of KO group were higher than those of WT group, regardless of age, although the differences were not significant. Scale bars, 200 μm. Data are the mean ± SD. (b) Pathological differences between IRD and ARD. Inhibition of NP cell apoptosis and delayed progression of IRD by caspase-3 KO. While senescent cells accumulated, expression of extracellular matrix degradation enzymes enhanced, and ARD showed premature progression.

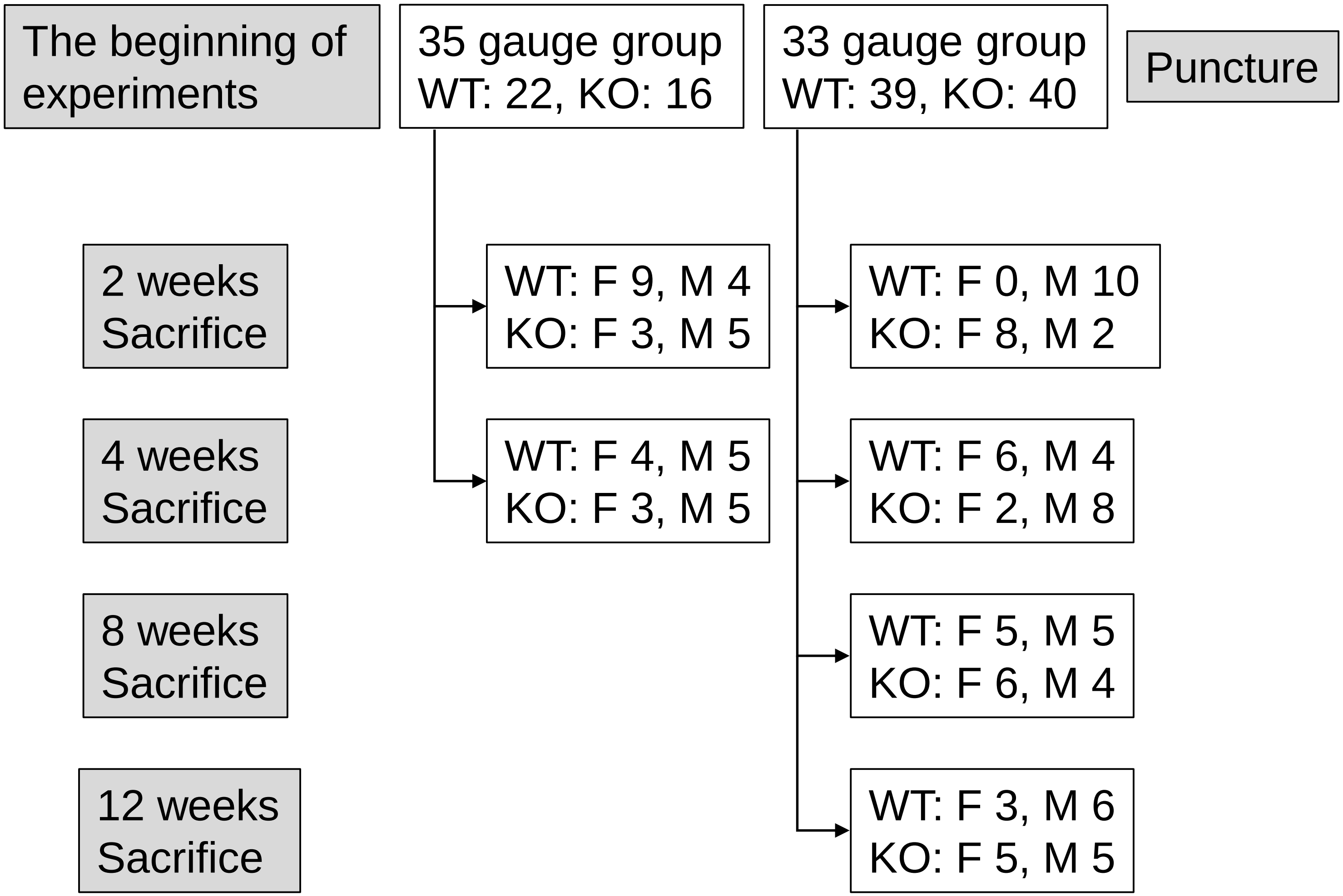

Supplementary Figure S4 Experimental flow diagram of injury-related IVD degeneration model. Numbers of wild-type (WT) and caspase-3 knockout (KO) mice of each sex are shown. F: female, M: male.

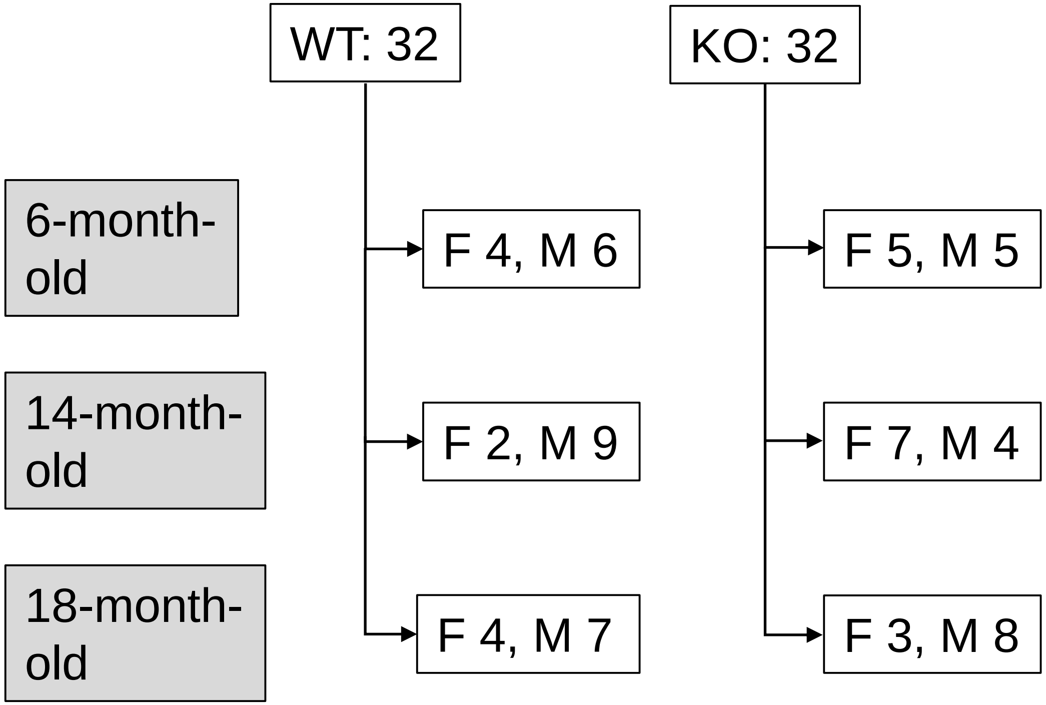

Supplementary Figure S5 Experimental flow diagram of age-related spontaneous IVD degeneration model. Numbers of wild-type (WT) and caspase-3 knockout (KO) mice of each sex are shown. F: female, M: male.
